# Control of dermatomyositis skin disease activity lags behind control of muscles disease activity during the early treatment stages of classic dermatomyositis: A retrospective, single‐centre study

**DOI:** 10.1002/ski2.357

**Published:** 2024-03-14

**Authors:** Heeruk Bhatt, Elizabeth M. Flatley, Kevin D. Cooper, Trine N. Jorgensen, Christine McDonald, Anthony P. Fernandez

**Affiliations:** ^1^ Cleveland Clinic Lerner College of Medicine at Case Western Reserve University Cleveland Ohio USA; ^2^ Robert Wood Johnson Medical School Rutgers University New Brunswick New Jersey USA; ^3^ Department of Dermatology Cleveland Clinic Cleveland Ohio USA; ^4^ Department of Dermatology Case Western Reserve University and University Hospitals Cleveland Medical Center Cleveland Ohio USA; ^5^ Department of Inflammation and Immunity Lerner Research Institute Cleveland Clinic Cleveland Ohio USA; ^6^ Department of Pathology Cleveland Clinic Cleveland Ohio USA

## Abstract

This study aimed to retrospectively identify differences in relative control of cutaneous and muscular disease activity in adult dermatomyositis (DM) patients at a single, tertiary care centre following initiation of diagnosis and treatment. Our results demonstrated a significantly lower complete treatment response rate of skin disease compared to muscular disease at 6‐months and persistent but not statistically significant lower skin disease response at 12 months. These results suggest DM skin disease activity may be more refractory to treatment than muscle disease activity, especially in the early disease phase.

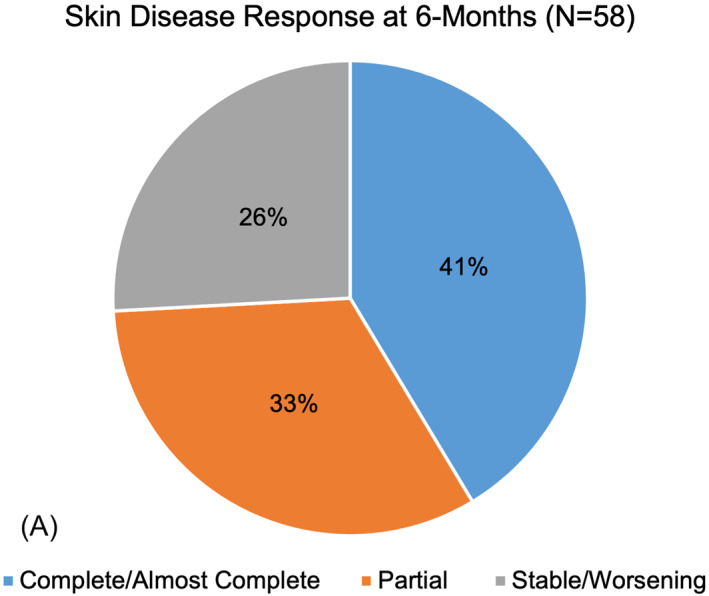

Dear Editor,

Classic dermatomyositis (DM) is an autoimmune disease characterised by active inflammation affecting muscle and skin.^1^, ^2^ Studies in paediatric populations have reported increased persistence of DM skin disease activity compared to muscle disease activity.^3^, ^4^, ^5^ In adults, one study revealed only 38% of DM patients achieved clinical remission of skin disease within 3 years.^6^ However, there is a paucity of data in adult DM populations concerning relative persistence of skin versus muscle disease activity. We explored for differences in relative persistence of skin versus muscle disease activity in a cohort of adult DM patients seen at a single, tertiary care centre. Our hypothesis was that DM skin disease activity is more persistent and refractory to treatment than muscle disease activity.

We retrospectively conducted a review of patients with classic DM who were prospectively incorporated into an IRB‐approved departmental DM Registry. All patients had been seen by a dermatologist and met EULAR diagnostic criteria for classic DM.^7^ Data concerning cutaneous and musculoskeletal disease activity during clinic visits at baseline (time of DM diagnosis), 6‐months (interval of 4–8 months), and 12‐months (interval of 9–15 months) was extracted from patient charts. We excluded patients without classic DM, patients diagnosed with DM prior to their first recorded chart record, and patients without adequate documentation to assess skin or muscle activity at above intervals.

Therapeutic response of cutaneous DM disease activity at 6‐ and 12‐months was assessed based on clinician documentation, cutaneous disease area and severity index activity scores, and photographs (when available).^8^ Therapeutic response of DM muscle disease activity was assessed based on clinician documentation, muscle exam scores and serum levels of creatine kinase and aldolase. For both skin and muscle disease activity, treatment response at 6‐month and 12‐month time points was categorised as complete/almost complete, partial, or stable/worsening based on prior research.^9^ At the 6‐month timepoint, treatment regimen changes were also noted for sub‐analyses, with patients being categorised as continuing stable treatment, treatment escalation (increased dose(s) of current medication or addition/change of systemic medications), or treatment de‐escalation (decreased doses/discontinuation of systemic medications). Fischer's exact test was used to determine statistical significance, with a *p*‐value <0.05 set as the significance threshold.

Our cohort was comprised of 66 patients with classic DM (median age 50 years; 83% female). Of these 66 patients, skin disease response at 6‐months was available for 58 patients, muscle disease response at 6‐months was available for 57 patients, and 52 patients had information pertaining to skin and muscle disease response at 12‐months. Myositis‐associated antibodies were identified in 44% of patients. At the 6‐month time point, a significant (*p* = 0.0013) difference was observed in the complete response rate of cutaneous disease (41%) compared to muscle disease (72%) (Figure 1a,b). At 12‐months, the difference in complete response rate of cutaneous disease (65%) remained lower than complete response rate of muscle disease (79%), but the difference was no longer significant (*p* = 0.1891; Figure 1c,d).

**FIGURE 1:**
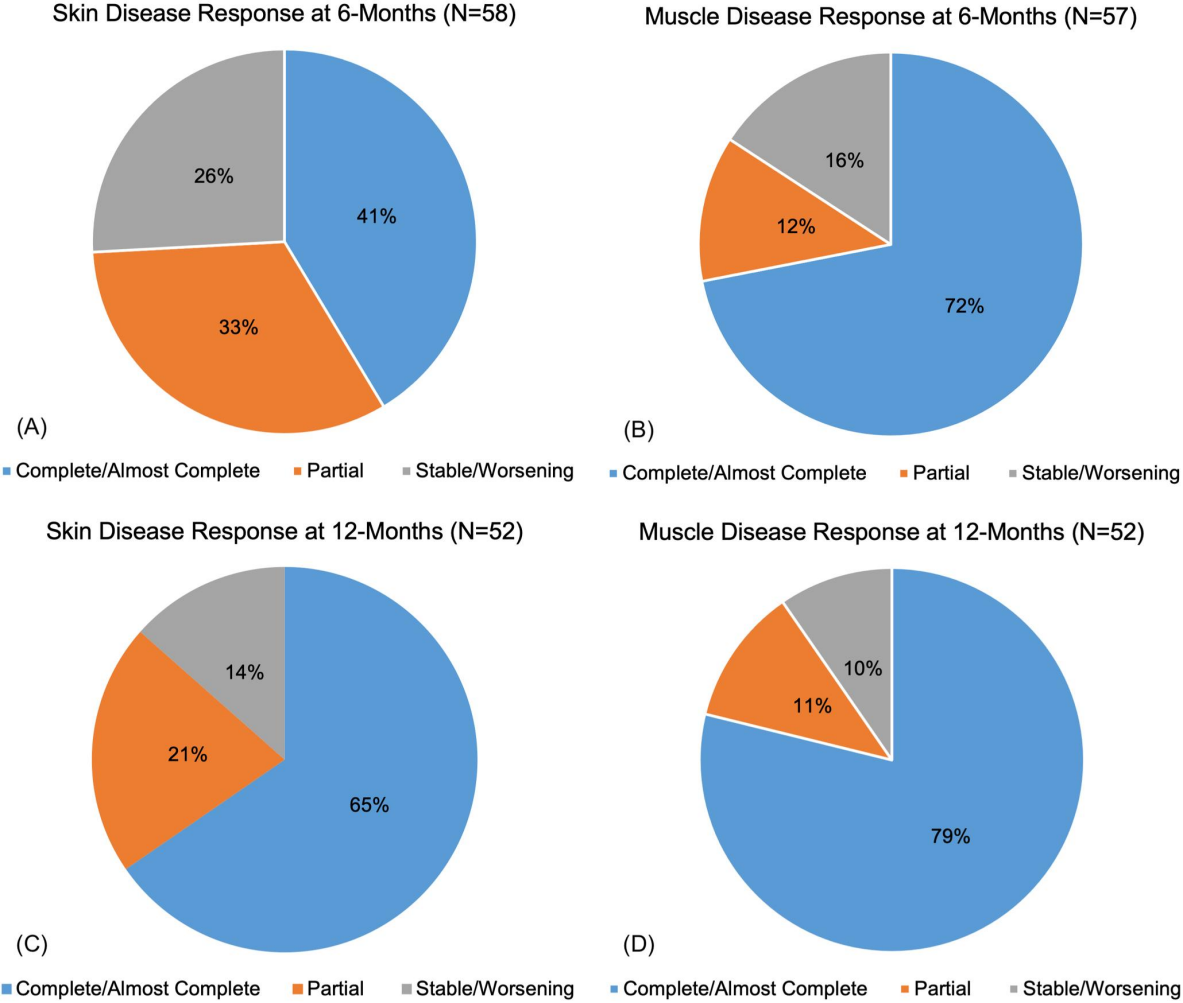
Comparing skin and muscle disease response at 6‐ and 12‐month time points. Response of skin (a) and muscle (b) to treatment evaluated at 6‐months (interval 4–8 months). Disease response was categorised as either complete/almost complete, partial, or stable/worsening. Fischer's exact test showed a significant difference (*p* = 0.0013) between complete response and non‐complete response (partial or stable/worsening) at the 6‐month time point. Repeat evaluation of response of skin (c) and muscle (d) disease to treatment was performed at 12‐months (interval 9–15 months). Calculation of Fischer's exact score demonstrated a non‐significant (*p* = 0.1891) difference between complete response and non‐complete response at 12‐months.

Patients whose treatment regimens were either stably maintained or deescalated at the 6‐month time point had non‐significantly lower cutaneous disease complete response rates (53% and 65%, respectively) at 12 months compared to muscle disease complete response rates (76% and 70%, respectively). Conversely, treatment escalation at 6 months resulted in equivalent (86%) complete response rates for cutaneous and muscle disease. Direct comparison of cutaneous disease response at 12‐months demonstrated a non‐significantly (*p* = 0.3932) lower complete response rate in the treatment de‐escalation group (65%) compared to the treatment escalation group (86%). There was also a non‐significant lower complete response rate for muscle disease at 12‐months in treatment de‐escalation (70%) as compared to treatment escalation (86%) cohorts (*p* = 0.6378).

In summary, cutaneous DM disease activity was significantly less likely to be completely controlled 6 months after diagnosis and treatment initiation compared to muscle disease in our cohort. Although the percentage of patients whose cutaneous disease was completely controlled at 12 months remained lower than the percentage whose muscle disease was completely controlled, the difference was non‐significant. Overall, our results suggest active DM skin inflammation may be as responsive to treatment as active muscle inflammation but may require longer periods of time and/or more aggressive treatment regimens to display this responsiveness. Whether this is related directly to relative tissue responsiveness of systemic medications or other factors is unclear. Our study has numerous limitations, including its retrospective nature, lack of objective skin/muscle disease assessments in numerous patients, and variability in actual assessment time points utilised for 6‐month and 12‐month analyses and in the physician performing evaluations from patient to patient. Additional limitations include variable patient exposure to DM‐exacerbating environmental factors, including UV light. Strengths include a well‐defined cohort who were all followed from point of diagnosis and documentation by physicians well‐experienced in treating and assessing skin and muscle disease in DM patients.

Future prospective research with larger cohorts is warranted to better assess differences between skin and muscle disease resolution.

## CONFLICT OF INTEREST STATEMENT

EMF, HB, KC, TNJ, CM: No conflicts of interest or financial disclosure. APF: investigator for Pfizer, Corbus, Mallinckrodt, Alexion, Priovant and Novartis and receives personal research support from Mallinckrodt and Novartis; honorarium from AbbVie, BMS, Biogen, Novartis, and UCB for consulting and advisory board participation, and honorarium from AbbVie, BMS, Kyowa Kirin, and Mallinckrodt for teaching and speaking (non‐promotional).

## AUTHOR CONTRIBUTIONS

**Heeruk Bhatt**: Conceptualization (lead); data curation (equal); formal analysis (lead); investigation (lead); methodology (lead); writing—original draft (equal); writing—review and editing (equal). **Elizabeth M. Flatley**: Data curation (equal); formal analysis (equal); writing—original draft (lead); writing—review and editing (lead). **Kevin D. Cooper**: Conceptualization (equal); data curation (equal); methodology (equal); project administration (equal); writing—review and editing (supporting). **Trine N. Jorgensen**: Conceptualization (equal); data curation (equal); methodology (equal); project administration (equal); writing—review and editing (supporting). **Christine McDonald**: Conceptualization (equal); data curation (equal); methodology (equal); project administration (equal); writing—review and editing (supporting). **Anthony P. Fernandez**: Conceptualization (equal); data curation (equal); investigation (equal); methodology (equal); project administration (equal); supervision (lead); validation (equal); writing—original draft (equal); writing—review and editing (equal).

## FUNDING INFORMATION

This article received no specific grant from any funding agency in the public, commercial, or not‐for‐profit sectors.

## ETHICS STATEMENT

This study is approved by our IRB 13‐715. Patient consent was not required by our IRB for this study.

## Data Availability

Data available on request due to privacy/ethical restrictions.
